# On the Microstructure and Isothermal Oxidation at 800, 1200, and 1300 °C of the Al-25.5Nb-6Cr-0.5Hf (at %) Alloy

**DOI:** 10.3390/ma12162531

**Published:** 2019-08-08

**Authors:** Ofelia Hernández-Negrete, Panos Tsakiropoulos

**Affiliations:** Department of Materials Science and Engineering, Sir Robert Hadfield Building, The University of Sheffield, Mappin Street, Sheffield S1 3JD, UK

**Keywords:** coatings, intermetallics, aluminides, pest oxidation, high temperature oxidation, Nb-silicide-based alloys, high entropy alloys, complex concentrated alloys

## Abstract

Nb-silicide-based alloys have the potential to replace Ni-based superalloys in future aero engines to enable the latter to meet environmental and performance targets. These new alloys, like the Ni-based superalloys that are currently used, will require environmental protection with a coating system that should be chemically compatible with the substrate. A challenge for alloy development is to discover αAl_2_O_3_ scale forming coating alloys and in particular to find out whether such alloys could be “compatible” with other coating alloys for environmental coating systems for the Nb-silicide-based alloys. This paper focuses on these challenges. The alloy Al-25.5Nb-6Cr-0.5Hf (at %) was studied in the cast and heat-treated (1400 °C) conditions and after isothermal oxidation for 100 h in air at 800, 1200 and 1300 °C. The microstructure consisted of the alloyed NbAl_3_ and C14-NbCr_2_ compounds, both of which were stable at least up to 1400 °C, a eutectic of the two compounds and very small volume fractions of (Cr,Al,Nb)_ss_ and HfO_2_. The prior eutectic microstructure was stable at T ≤ 1200 °C and the solid solution was not stable at T < 1200 °C. At 800 °C the alloy did not pest, but exhibited external and internal oxidation, with AlNbO_4_, CrNbAlO_4_, and αAl_2_O_3_ in the former and deeper oxidation along the NbAl_3_/Laves phase boundaries in the latter At 1200 and 1300 °C there was only external oxidation and the scale consisted of two layers, the outer was (Al,Cr)NbO_4_ intermixed with αAl_2_O_3_ and the inner was continuous αAl_2_O_3_. At all three oxidation temperatures, no Nb_2_Al was observed below the alloy/scale interface and Hf acted as a reactive element forming HfO_2_ that enhanced the adhesion of the scale. The alloy exhibited good correlations with αAl_2_O_3_ scale forming silicide and silicide + aluminide intermetallic alloys in maps of the parameters δ (related to atomic size), Δχ (related to electronegativity), and VEC (number of valence electrons per atom filled into the valence band) that should assist the design of bond coats that do not pest and form αAl_2_O_3_ in their scales.

## 1. Introduction

Single crystal Ni-based superalloys are the current state of the art high-temperature structural materials in gas turbine engines. These materials are limited by the melting point of Ni [1]. There is a need for lower density materials that can operate at higher temperatures than Ni-based superalloys to achieve higher thrust-to-weight ratio in aircraft engines and other propulsion systems [2,3]. In gas turbine engines a reduction in turbine blade weight will have a cascading effect throughout the entire rotor (disk, hub, and shaft) and non-rotating support structures [3]. For many ultra-high-temperature structural applications in areas of propulsion and energy conversion, high-strength and creep-resistant refractory metal alloys are the materials of choice.

Intermetallics are believed to present more possibilities than conventional refractory metal alloys and ceramic materials. They can be cooled to limit the maximum material temperature owing to their thermal conductivities and they also offer the opportunity to include oxide forming elements like Al, Si, and Cr for improved environmental resistance. The creep properties of some binary intermetallics, for example the tetragonal Nb_5_Si_3_ [4], indicate that alloyed intermetallics could have creep resistance comparable to refractory metal alloys. The monolithic intermetallics are brittle at low temperatures and have a ductile to brittle transition temperature (DBTT) that is lower than the softening temperature of alumina (for example the DBTT of NiAl, MoSi_2_, Nb_3_Al, and NbAl_3_ respectively, is about 400, 1100, 900, and 830 °C compared with 1370 °C of alumina [5,6,7,8]), and these intermetallics are ductile at the use temperature. The lack of ductility of intermetallics at room temperature necessitates a composite approach to impart sufficient damage tolerance at low temperatures [9].

Nb-silicide in-situ composites or Nb-silicide-based alloys have: (a) densities lower than Ni-based superalloys, (b) liquidus temperatures above 1900 °C, (c) microstructures that consist of (i) bcc Nb solid solution(s) [10], (ii) tetragonal Nb_5_Si_3_, with/without (iii) other intermetallics such as tetragonal Nb_3_Si, hexagonal C14-NbCr_2_ Laves and Nb_5_Si_3_ and cubic A15-Nb_3_X (X = Al, Ge, Si, Sn) [11,12,13,14,15], with/without (iv) binary or ternary eutectics [16,17,18,19], and (1) can offer a balance of mechanical, ballistic, and environmental properties, and (2) have the potential to replace Ni-based superalloys in propulsion systems. The oxidation resistance of Nb-silicide-based alloys depends on alloying additions. These alloys are not alumina or chromia formers (see below). The improved oxidation behaviour is attributed to niobate and NbAlO_4_ and/or NbCrO_4_ rutile oxide structures that make up their scales [14]. Like the Ni-based superalloys, these new materials will require environmentally resistant coatings [20]. Most likely, the latter will be of the bond coat (BC)/thermally grown oxide (TGO)/top coat (TC) type, where the BC could be layered multi-material or functionally gradient material [20,21]. The environmentally resistant coating would be applied to an alloy (substrate) with oxidation resistance. It is desirable for the BC to form αAl_2_O_3_ as TGO.

In Ni-based superalloys, increased Cr concentration is weakly correlated with improved cyclic oxidation resistance that typically occurs for Cr above 5 wt % [22] and additions of about 6 wt % Al are good enough for inherent oxidation resistance. The BC alloys for Ni-based superalloys are richer in Al and form αAl_2_O_3_ as TGO (MCrAl type BCs typically have less than about 15 wt % Al (25 at %) and rely on Cr to promote exclusive Al_2_O_3_ formation) [23]. Nb-silicide-based alloys do not form alumina or chromia scales owing to their Al and Cr concentrations that are restricted to low values because (a) both elements increase significantly the DBTT of the Nb_ss_ above ~50 °C [24], (b) high Cr concentrations can cause liquation at high temperatures [25], and (c) high Al concentrations stabilise the Nb_3_Al which pests and does not form protective Al_2_O_3_ scales at high temperatures [26].

The developments of (a) Nb-silicide based alloys and (b) of coatings suitable for these alloys have been instructed by engineering judgement and have suffered from the shortage of reliable and wide-ranging thermodynamic data. The alloy design methodology NICE (Niobium Intermetallic Composite Elaboration) was created [14] and advanced to achieve alloy density reductions, microstructural stability, oxidation resistance, and high-temperature strength and creep resistance for Nb-silicide-based alloys. The foundations of NICE are the alloying behaviour of Nb-silicide-based alloys [27] and their key phases [4,15,18]. The parameters δ (related to atomic size), Δχ (related to electronegativity), and VEC (the number of valence electrons per atom filled into the valence band) are key alloy design parameters in NICE. The latter offers good predictive capability within the compositional design space and provides guidance for optimisation experiments to achieve targeted property goals [14].

**Figure 1:**
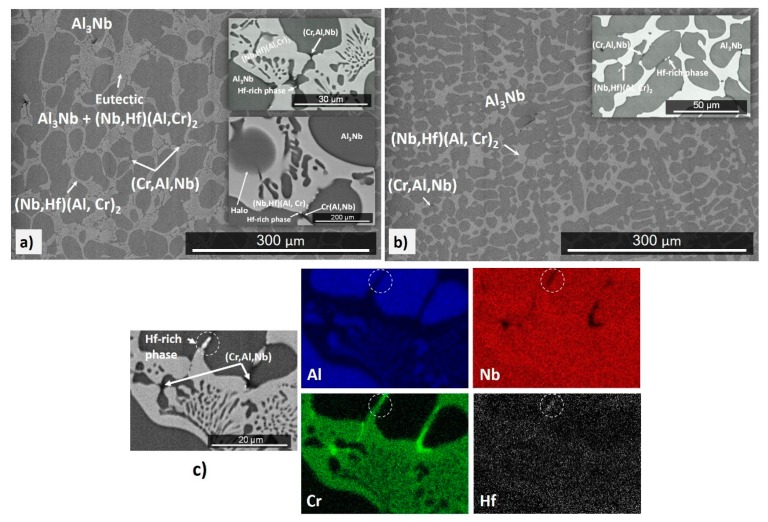
Back scatter electron (BSE) images (**a**) and (**b**), and (**c**) X-ray maps of the microstructure of the alloy OHC3-AC, (**a**) bulk, X190, (**b**) bottom, X200, (**c**) BSE image (X2000). In (**c**) the encircled areas correspond to the Hf-rich particle.

The capabilities of NICE for the design of coating alloys were demonstrated recently with the design and selection of intermetallic alloys of the Al-Hf-Nb-Ti-Si system that do not suffer from catastrophic pest oxidation and form alumina scales at 1200 °C [21,28]. The microstructures of these alloys consisted of hexagonal 5-3 silicides, the Ti_5_Si_4_ and TiSi silicides, and Al-rich TiAl and Ti-rich DO_22_-TMAl_3_ aluminides. The latter were the Al reservoirs for alumina formation in the scale of the alloys. The alloys of the aforementioned system were “well-matched” with non-pesting intermetallic alloys of the Al-Cr-Fe-Nb-Si-Ti system in maps of the parameters δ, Δχ, and VEC, see Figure 1 in reference [29] and Figure 23 in reference [30].

DO_22_-NbAl_3_ formed in a silicide/aluminide coating on a Nb-silicide-based substrate that was prepared by two-step pack cementation (siliconizing and then aluminising). A mixed oxide scale formed in air at 1250 °C after 50 h that consisted of an outer layer of Al_2_O_3_ with dispersed TiO_2_ above a glass-like Si-rich oxide layer with dispersed Al_2_TiO_5_, TiO_2_, and NbCrO_4_ [31]. We were interested in discovering intermetallic alloys that could be candidate bond coat alloys for environmentally resistant coatings for Nb-silicide-based alloys. The aforementioned maps do not include alloys with microstructures consisting of DO_22_-NbAl_3_ and C14-NbCr_2_ Laves phases and no silicides. What would be the oxidation of an alloy with these compounds in its microstructure? Would it pest? Would it form continuous alumina? Would alumina scale formation occur at temperatures lower than 1200 °C? Would the alloy form a layered microstructure? Would the alloy be “compatible” with silicide and silicide + aluminide coating alloys? The research presented in this paper was motivated by the need to answer these questions. Before we discuss how the alloy of this study was selected, we would like to briefly comment on the oxidation of the above two intermetallics so that the challenges for the research presented in this paper become clear.

In the Al-Nb binary system, the NbAl_3_ has a narrow solubility range (74.5 to 75.5 at % Al [32]), is the lightest aluminide (density ρ = 4.54 g/cm^3^), has the slowest oxidation rate of all Nb-Al compounds at 1200 °C, but suffers from pest oxidation in the temperature range 700 to 900 °C [33]. The ratio G/B (G = shear modulus, B = bulk modulus) of the NbAl_3_, Nb_2_Al, and Nb_3_Al aluminides respectively, is 0.804, 0.448, and 0.38, meaning that the NbAl_3_ is the most brittle compound in the Nb-Al system [34]. Both NbAl_3_ and Nb_2_Al are chemically compatible with Al_2_O_3_ as reinforcement [35]. At temperatures higher than the pest regime, the oxidation of NbAl_3_ depends on its Al concentration [36]. The Al-rich NbAl_3_ forms a continuous αAl_2_O_3_ scale at 1200 °C. The Al-lean Al_3_Nb does not form an exclusive αAl_2_O_3_ layer. An inner layer of alumina forms on the NbAl_3_ adjacent to the metal/oxide interface and an NbAlO_4_ outer layer forms at the oxide/gas interface. The parabolic rate constant for oxidation in air at 1200 °C is about 3 × 10^−10^ g^2^/cm^3^·s [37]. Depletion of Al at the metal/scale interface leads to the formation of Nb_2_Al. The latter is an open compound with a wide range of solubility and forms NbAlO_4_ in its scale [37]. The diffusivity of Al through this phase is not sufficient to maintain the flux required to form alumina as the layer thickness of the phase increases and the phase itself does not have sufficient Al to form alumina. The result is a breakaway effect in which both Nb and Al are oxidised forming NbAlO_4_ under the original alumina scale with accelerated kinetics. This continues until the Nb_2_Al layer is consumed, at which time the cycle will repeat, and a new alumina layer will be formed, cutting off growth of the NbAlO_4_ scale and reforming the Nb_2_Al at the interface. Alumina can form in air on NbAl_3_ at 1350 °C where layers of α Al_2_O_3_ and NbAlO_4_ were evident after 1 h but the growth of a protective alumina scale was not sustained [38].

The NbCr_2_ Laves phase has a higher melting point and is denser than NbAl_3_ (1770 °C and 7.66 g/cm^3^), its DBTT is similar to Nb_2_Al (around 0.65 T_m_, where T_m_ is melting temperature), has toughness less than 2 MPam^1/2^, and bulk modulus 200 GPa compared with 153 GPa for Nb [39,40]. At 1100 °C, the NbCr_2_ exhibits a transition to rapid oxidation after 5 to 10 h in humid air [41] and forms NbCrO_4_ and Cr_2_O_3_, the same as at 1200 °C. This is analogous to the oxidation products found on NbAl_3_ but the Cr_2_O_3_ oxide is located at the oxide/gas interface rather than the metal/oxide interface. There is also nitridation below the scale (about 100 to 150 μm deep at 1100 °C [41]). The nitridation follows the underlying alloy microstructure which is consumed in place (in situ internal oxidation). The parabolic rate constant of NbCr_2_ in air at 1200 °C is 2.9 × 10^−9^ g^2^/cm^4^·s [37]. Cr_2_O_3_ scales are protective up to 1000–1100 °C (lower in the presence of high flow-rates and/or water vapour) due to the CrO_3_ volatility [42]. Compared with other Laves phases, only the NbCr_2_ was less affected by oxidation when mechanical properties were evaluated at temperatures up to 1400 °C [40].

A challenge for the design of an oxidation-resistant alloy with NbAl_3_ and NbCr_2_ as its key phases is to enhance its oxidation behaviour compared with that of each of its constituents. The selection of the alloy composition must take into account the effects of alloying on processing and on the oxidation of these two phases. A reduction of the oxidation rate of the NbAl_3_ at 1200 °C has been achieved with additions of Cr and Y [43,44]. The solubility of Al in the C14-NbCr_2_ Laves phase is significant (≈ 45 at % Al), the Nb(Cr, Al)_2_ is stabilized to lower temperatures, its melting temperature is decreased with Al [45], and the oxidation of Nb(Cr, Al)_2_ and the adhesion of the scale are better compared with the unalloyed Laves phase [46].

The structure of the paper is as follows. Next, we discuss how the alloy composition was selected. This section is followed by a brief description of the experimental techniques that we used, which essentially were the same as discussed in reference [30]. The results for the cast and heat-treated microstructures of the alloy and its isothermal oxidation at 800, 1200, and 1300 °C are then presented. In the discussion we deliberate on the microstructures of the alloy and the chemical compositions of the compounds before we consider the oxidation of the alloy. The latter is then compared with the alumina forming Nb-Ti-Si-Al-Hf and Si-Nb-Al-Cr-Ti alloys in the maps of the parameters δ, Δχ, and VEC.

## 2. Alloy Selection

The maps in Figure 1 in reference [29] and Figure 23 in reference [30] were produced with data for non-pesting and alumina-forming intermetallic alloys with Al, Cr, Fe, Hf, Nb, Si, and Ti alloying elements and no stable solid solution. Some of these alloys satisfy the “standard” definition of High Entropy Alloys (HEAs) and could be considered to be Multi-Principle Element Alloys (MPEAs) or Complex Concentrated Alloys (CCAs) [21,28]. In the research presented in this paper we wished to select an alloy that met the following requirements. The alloy should: (a) be an Al-rich intermetallic alloy with no Fe, Si, and Ti additions, (b) have Hf as a reactive element, (c) have microstructure consisting of primary DO_22_-NbAl_3_ tri-aluminide and C14-NbCr_2_ Laves phase with no stable solid solution, (d) not suffer from incipient melting below 1400 °C, (e) not pest, (f) form continuous alumina scale, (g) be located in the compositional design space defined by the parameters δ, Δχ, and VEC in the aforementioned maps, and (h) not be constrained to be an HEA, MPEA, or CCA alloy. Requirements (a) and (b) confine the alloy to belong in the Al-Cr-Hf-Nb system. The formation of a layered microstructure in the alloy is desirable (see below). Let us now consider the above requirements.

### 2.1. Why Al-Rich, NbAl_3_-Based Alloy?

Research has demonstrated that Nb-Al alloys must contain Al above 32 at % and aluminides in order to form an alumina scale [47]. Aluminium concentrations in Nb-Al alloys that are higher than this “critical” concentration can stabilise DO_22_-NbAl_3_ (Hf (like Nb and Ti) forms DO_22_ tri-aluminide but Fe forms mC102-FeAl_3_). The alloying of NbAl_3_ with Hf results in an increase of density but the effect on elastic constants and the G/B ratio is very small [48]. The DO_22_-NbAl_3_ has a higher melting point than the DO_22_-TiAl_3_. The latter was formed (together with Al rich TiAl) in the alumina-forming MG series alloys (14.5Nb-27Si-22.5Ti-32.5Al-3.5Hf (MG5), 13.5Nb-23Si-23Ti-37Al-3.5Hf (MG5), 13Nb-24Si-24Ti-35Al-4Hf (MG7)) that had outstanding oxidation resistance at 800 and 1200 °C owing to the high-volume fraction of aluminides [21,28]. In their un-alloyed form, TiAl and TiAl_3_ have melting temperatures about 1460 and 1325 °C and densities of 3.91 and 3.4 g/cm^3^ respectively, compared with the higher melting temperature (1710 °C [49]) of the denser (4.54 g/cm^3^) NbAl_3_, which is higher than that (1640 °C) of the denser (5.86 g/cm^3^) NiAl used in coatings for Ni-based superalloys.

Relying on Ti-based aluminides restricts the upper use temperature of an intermetallic alloy as a coating material. We are interested in stable microstructures and alumina scales for oxidation resistance up to 1400 °C because alumina scales offer the potential to protect Nb alloys from oxidation for long times at temperatures up to 1400 °C, where Nb alloys can be used safely without concern for oxide melting and catastrophic oxidation if breakaway occurs [47].

Aluminium, like Si, stabilises the oxidation-resistant C14-NbCr_2_ Laves phase [50], which is desirable for this study. Aluminium diffuses faster than Nb in NbAl_3_ [51]. A high Al concentration may increase the diffusivity of Al in Nb-Al alloys at high temperatures, a requirement from Wagner’s theory of oxidation [52] for the formation of an external alumina scale (see below). However, the high concentration of Al that is needed to have an Nb-Al alloy that forms an external Al_2_O_3_ scale necessitates the selection of specific alloying additions (third element effect), like Cr and Hf. This will be discussed next. A high Al concentration in an intermetallic-based alloy may also lead to the formation of a layered microstructure, as was the case in the alloy MG7 [21]. This will be discussed later on in Section 2.6.

### 2.2. Why Cr, Hf, and Laves Phase?

The high temperature oxidation resistance of alloys requires alloying additions that enable the formation of an external continuous layer of a stable oxide that separates the alloy from the oxidising (corrosive) environment. This is often referred to as selective oxidation and requires that the oxide of the added element be the most stable oxide of any of the other major components in the alloy. Wagner’s theory [52] indicates that for the transition from internal to external oxidation of Nb-Al alloys, alloying additions must be chosen that decrease the oxygen solubility and diffusivity and increase the diffusivity of Al in the alloy. Solutes like Hf and Cr respectively, with more negative enthalpies of formation of their oxides and smaller atomic radii than Nb, may decrease the diffusivity of oxygen by providing attractive traps [47,53]. Chromium, as one of the elements that increase the electron concentration in Nb, may also decrease the oxygen solubility by increasing the activity of oxygen [47]. Hafnium may also decrease the oxygen solubility through the gettering effect.

Both Hf and Cr not only can promote selective oxidation of Al in Nb-Al alloys but they also form/participate in Laves phases (HfCr_2_, NbCr_2_, (Nb,Hf)Cr_2_ [54]). Research has confirmed that Cr is the most effective ternary addition in NbAl_3_ favouring the selective oxidation of Al. For example, the addition of 6 at % Cr in the alloy Nb-70Al-6Cr resulted in the formation of a Cr-rich phase in-between NbAl_3_ grains, there was no internal oxidation, an external scale that consisted of continuous αAl_2_O_3_ below NbAlO_4_ was formed in air at 1200 °C where the parabolic rate constant was 6.1 × 10^−11^ g^2^/cm^4^·s [43] compared with 3 × 10^−10^ g^2^/cm^4^·s for NbAl_3_ [37].

### 2.3. Why Hf as a Reactive Element and not Y?

In addition to the fact that Y is not in the compositional design space of the alloy of this study (see above), there are other reasons for not using Y. For some time, a conventional means of maintaining adherent alumina scales on the surface of heat resistant alloys has been the addition of small concentrations of reactive elements such as < 1 wt % of Y, Zr, Hf, La, Sc or other group 3 and 4 elements including the lanthanide and actinide series. The amount and uniformity (homogeneous distribution) of the reactive element is crucial for optimum scale adhesion. There is no single optimum level of reactive element addition that applies to all reactive elements in all host alloys. Yttrium has been the most popular in superalloys. Small additions of Hf significantly enhance the adhesion of Al_2_O_3_ scales in alumina-forming alloys [55]. Hafnium and Zr are as effective in MCrAl and Ni-Al systems. Obtaining the targeted level of retained Y in a refractory metal intermetallic alloy can be challenging [56], as has been the case with Ni-based superalloys. In single crystal Ni-based superalloys, enrichment in Y can cause some incipient melting during solution treatment resulting in Y-rich particles and porosity. Furthermore, when Y was added as a reactive element in the NbAl_3_-based alloys Nb-68Al-7Cr-0.5Y and Nb-70.8Al-5.1Cr-0.5Y (at %), the microstructure consisted of NbAl_3_ with Y- and Cr-rich (not Laves) phases in- between the NbAl_3_ grains [43,44]. The scale that formed on the former alloy in air at 1200 °C consisted of external continuous αAl_2_O_3_ that formed below NbAlO_4_. However, at 1400 °C there was deep penetration of oxygen and the scale consisted of αAl_2_O_3_ and several unknown phases [43].

### 2.4. Why no Fe, Si, and Ti?

Iron was not considered as an alloying addition because of concerns about incipient melting below 1200 °C and the nucleation of C14 Laves phase on NbAl_3_. Indeed, Fe forms the low-melting (1160 °C) mC102 FeAl_3_ [32]. Furthermore, despite the fact that Fe forms the C14-NbFe_2_ Laves phase and, with Al, stabilises the C14-Nb(Al,Fe)_2_ and Hf(Al,Fe)_2_ [57,58], the former ternary Laves phase had low nucleation potency on the primary NbAl_3_ [7] in the Nb-67Al-9Fe (at %) alloy, in which the FeAl_2_ was also formed in arc-melted ingots in addition to the Laves phase.

Titanium and Si may decrease the oxygen solubility in Nb-Al alloys [43]. However, when each of these elements was added to NbAl_3_-based alloys the oxidation resistance decreased. The alloy Nb-70Al-6Si (at %) formed a continuous Al_2_O_3_ scale in air at 1200 °C but there was internal oxidation of Al along Si-rich phases that were formed in-between the NbAl_3_ grains and the parabolic rate constant was k_p_ = 1.25 × 10^−10^ g^2^/cm^4^·s. Also, in air and at 1200 °C the oxidation of Nb-70Al-6Ti (at %), which formed Al_2_O_3_ below NbAlO_4_, was worse (k_p_ = 3.1 × 10^−9^ g^2^/cm^4^·s [43]). The k_p_ values of the alloys with Si or Ti addition were higher respectively, by one and two orders of magnitude compared with the alloy Nb-70Al-6Cr (see above). Furthermore, TiO_2_ rutile can be a (sometimes significant) component of the oxidation products on Ti and Al containing intermetallic alloys. Cationic diffusivity is considerably higher in rutile than in alumina and rutile can destruct the formation of a continuous alumina scale.

### 2.5. Why no Stable Solid Solution?

In the Al-Cr binary alloy system, the Cr solid solution Cr(Al) dissolves a maximum of 45 at % Al at 1320 °C. In the Nb-Cr binary alloy system the NbCr_2_ forms eutectics with the Nb-rich Nb(Cr) and Cr-rich Cr(Nb) solid solutions. The Nb(Cr) is susceptible to oxygen embrittlement and the Cr(X) can be brittle at room temperature and embrittled by nitrogen at high temperatures. At 1100 °C, the oxidation scale formed on the Cr(Nb) + NbCr_2_ eutectic was similar to that of the Laves phase, but the mass change was higher and there was nitridation underneath the scale [41].

In ternary Al-Nb-Cr alloys with Al > 60 at % the primary phase is the NbAl_3_, and in such Al-rich alloys the DO_22_ intermetallic is surrounded by the C14-NbCr_2_ Laves phase [45]. There is a two-phase eutectic C14-NbCr_2_ + NbAl_3_ at about 1520 °C, and depending on the alloy composition, as the composition of the liquid runs along the eutectic valley, it can give either a ternary eutectic C14-NbCr_2_ + NbAl_3_ + Nb_2_Al (at about 1502 °C) or a three-phase microstructure C14-NbCr_2_ + NbAl_3_ + Cr_ss_ at about 1352 °C [45]. The formation of Nb_2_Al and a stable solid solution are not desirable in our alloy. Would the addition of Hf destabilise these undesirable phases?

We now turn our attention to the possibility of forming a Zone A and/or layered microstructure.

### 2.6. Why a Zone A Microstructure?

Research in our group [21,28,30] on intermetallic alloys that could be candidate bond coat alloys for environmentally resistant coatings for Nb-silicide-based alloys has discovered that a layered microstructure was formed in the alloy Nb-24Si-24Ti-35Al-4Hf (MG7 [21]) owing to transitions in the cast microstructure. In this alloy and the alloy Si-22Fe-12Cr-12Al-10Ti-5Nb (OHC2 [29]), an Al-rich zone was formed in the areas of the arc-melted buttons where the solidifying melt was in direct contact with the water-cooled copper crucible wall. This Al-rich zone has been called the Zone A microstructure. The average compositions (at %) of the Zone A in the alloys MG7 and OHC2 respectively, were 11Nb-12.8Si-20.5Ti-54Al-1.7Hf and 35.4Si-27.9Fe-25.5Al-7.1Cr-3Ti-1.1Nb. In the former alloy, the Zone A was dominated by Ti-rich tri-aluminide DO_22_-TMAl_3_ but in the latter alloy no tri-aluminides were formed. The high Al concentration in the Zone A of the alloy MG7 and the solidification conditions at the water-cooled copper crucible that acted as an effective heat sink had contributed to forming the Zone A [21].

Rapid quenching from the melt can lead to significant microstructural modification. Solidification under high cooling rates, such as those experienced by the melt in contact with the water-cooled copper crucible in arc melting, could involve solidification under bulk undercooling conditions [59,60,61]. Liquid Nb and Nb-based alloys can undercool up to 0.19T_m_^Nb^ (525 K) prior to nucleation at relatively slow cooling rates [62,63]. In melt-spun ribbons of binary hypereutectic and hypoeutectic Nb-Si alloys, with increasing distance from the chill surface, a transition in microstructure was observed between the wheel side (amorphous layer) and centre of the ribbons (fine-grained microstructure) [64]. In the cast microstructures of arc-melted Nb-silicide-based alloys with/without Al addition, a transition has been observed from an anomalous eutectic microstructure, which was formed in the melt that solidified in contact with the water cooled crucible, to anomalous + normal, and then to normal eutectic, and then to the microstructure seen in the bulk of the cast alloy [59,60,61]. In the literature, transitions between anomalous and normal eutectics have also been reported to occur in the unconstrained (i.e., free) solidification of bulk undercooled binary eutectic alloys [65,66,67,68]. Anomalous eutectic is formed when the melt undercooling exceeds a critical value [59,60,67,68]. When such transitions were observed in Nb-silicide-based alloys, the scale of the microstructure of the zone formed in contact with the water-cooled crucible was finer than the rest of the alloy and had simple metal and metalloid element content (e.g., Si + Ge, Si + Sn, Si + Al + Sn) significantly lower than the Al + Si concentration in the Zone A microstructure in the alloy MG7 [21] and lower than the Al concentration in the Zone A microstructure in the alloy OHC2 [29]. When the actual compositions of the alloys and their Zone A microstructures were considered, the ratio (Al + Si)_Zone A_/(Al + Si)_Alloy_ was the same for both alloys, respectively 1.12 for MG7 and 1.13 for OHC2, and the ratio (Al/Si)_Zone A_/(Al/Si)_Alloy_ was similar, respectively 2.79 for MG7 and 2.12 for OHC2. The hypothesis that Si played a key role in Zone A formation is supported by the strong macro-segregation of Si in the alloy MG7. One further reason for not including Si in the alloying elements of the alloy of this study was to test this hypothesis (see Section 6).

In the Nb-Al binary system there is a eutectic at 1590 °C and 59.5 at % Al [69]. Alloys near this eutectic composition can be undercooled significantly. Indeed, undercoolings up to 230 K have been reported for Nb-59.5Al (at %) in reference [69] and up to 240 K for Nb-60Al (at %) in reference [49]. The growth kinetics of highly undercooled bulk melts can display multi-step recalescence processes depending on melt composition and undercooling. For the eutectic alloy, Loser et al. [69] observed a transition from double to triple recalescence beyond a critical undercooling which increased with decreasing Al content (which would suggest that an increase in Al concentration could decrease the critical melt undercooling for such transitions). Furthermore, when an undercooled (230 K) hypoeutectic Nb-58Al (at %) alloy was quenched on a copper substrate, Loser et al. [69] reported (i) that a layer about 200 μm thick formed adjacent to the substrate, (ii) that the microstructure changed to an anomalous eutectic at distances above 200 μm from the substrate, and (iii) that the microstructure changed again towards the centre of the quenched sample, and that its scale depended primarily on the distance from the substrate. In undercooled Nb-60Al (at %), the Al concentration of the primary NbAl_3_ was in the range 69 to 74 at % [49], wider than that in the Nb-Al binary [32]. A change in morphology was also observed as the undercooling increased [49]. In summary, the limited literature about Al-rich eutectic or near eutectic Nb-Al alloys has indicated that: (a) the undercoolability of Nb-Al melts is high at high Al concentrations and increases with Al content, and (b) that the undercooling of such melts has three major effects, namely (1) microstructural refinement, (2) morphological and microstructural changes, and (3) widening of the solubility range of NbAl_3_. Would formation of a Zone A microstructure in an Al-rich quaternary Al-Cr-Hf-Nb alloy be possible?

Based on the available solidification, microstructure, oxidation, and phase equilibria data, the nominal alloy composition (at %) was selected to be 68Al-25.5Nb-6Cr-0.5Hf (OHC3). As was the case for the alloys MG5, MG6, MG7, OHC1, OHC2, and OHC5 [21,28,29,30], the alloy was not studied as a coating applied on a Nb-silicide-based substrate in order to eliminate the effects of substrate and coating processes on microstructures and isothermal oxidation.

## 3. Experimental

We used arc melting with a non-consumable tungsten electrode to prepare the alloy from pure elements (≥ 99.9 wt % purity) in a Ti gettered Ar atmosphere using a voltage of 50 V and a current of 650 A. Alloys prepared by arc melting usually exhibit severe macro-segregation and it is common practise to melt them a few times by turning the button in the copper crucible in order to chemically homogenise their microstructures as much as possible. In our case, the button was melted five times. For the heat treatment (1400 °C/100 h) we used an alumina tube furnace and a Ti gettered Ar atmosphere. The specimens were wrapped in Ta foil to minimize contamination by oxygen and were placed in an alumina crucible and cooled down in the furnace.

A metallographic preparation of specimens consisting of mounting in bakelite, grinding with SiC paper (from 120–1200 grit), and then to grade 4000, and cashmere cloth polishing with 1 µm diamond suspension was performed.

For the DSC experiments, a Rh/Pt furnace was used in the Netzsch STA F3 TG/DSC analyser (Netzch Gmbh, Waldkraiburg, Germany) with an Ar flow rate of 20 ml/min up to a temperature of 1600 °C. The isothermal oxidation of the as-cast alloys was studied at 800 °C, 1200 °C, and 1300 °C for 100 h using a Netzsch STA F3 TG/DSC analyser with a SiC furnace with air flow rate of 20 ml/min and with heating and cooling rates of 3 °C/min. Cubic specimens of size 3 mm × 3 mm × 3 mm and polished to 800 grit SiC finish were used for the thermo-gravimetry (TGA, SEM) experiments. The specimens for thermal analysis were selected from the bulk of the cast buttons.

The microstructures and oxide scales were characterised using scanning electron microscopy (SEM) and X-ray diffraction (XRD) and glancing angle XRD (GXRD). The (SEM), a Philips PSEM 500 SEM (Philips-Thermo Fisher Scientific, Hillsboro, OR, USA), Jeol JSM 6400 (Jeol, Tokyo, Japan), and Inspect F FEG SEM (Thermo Fisher Scientific, Hillsboro, OR, USA), were used. The back scatter electron (BSE) mode was used to study the microstructures with qualitative and quantitative chemical analysis performed with the use of the energy dispersive x-ray spectroscopy (EDS) analysis (Oxford Instruments, Abington, UK) of the alloy and phases (20 kV for image and EDS quantitative analysis and 20 kV and 15 kV respectively, for the X-ray elemental maps of the scale surface and cross-sections of oxidized specimens). EDS standardization was performed using specimens of high purity Nb, Cr, Al, Hf, and Co standards, that were polished to 1 μm finish. The EDS was calibrated prior to analysis with the Co standard. At least five large area analyses were performed in the top, bulk, and bottom of the button, and at least ten analyses were obtained from each phase with size ≥ 5 μm to determine actual compositions.

The Siemens D500 XRD diffractometer (Hilton brooks Ltd, Crew, UK) with CuKα radiation (λ = 1.540562 Å), 2θ from 20°–80° and a step size of 0.02° at 40 kV and 40 mA was used. For glancing angle XRD (GXRD), a Siemens D5000 diffractometer (HiltonBrooks Ltd, Crew, UK) with Cu Kα_1_ and Kα_2_ radiation (λ = 1.54178 Å), 2θ from 20°–70° and a step size of 0.02° at 40 kV and 40 mA was used. Peaks in the XRD diffractograms were identified by correlating data from the experiments with that from the JCPDS data (International Centre for Diffraction Data). The scan type used for GXRD was detector scan. Prior to GXRD experiments, the glancing angle was selected with the aid of the Absorb DX software which evaluates the X-ray penetration depth for particular glancing angle conditions.

## 4. Results

### 4.1. Cast and Heat-Treated Microstructures

**Figure 2:**
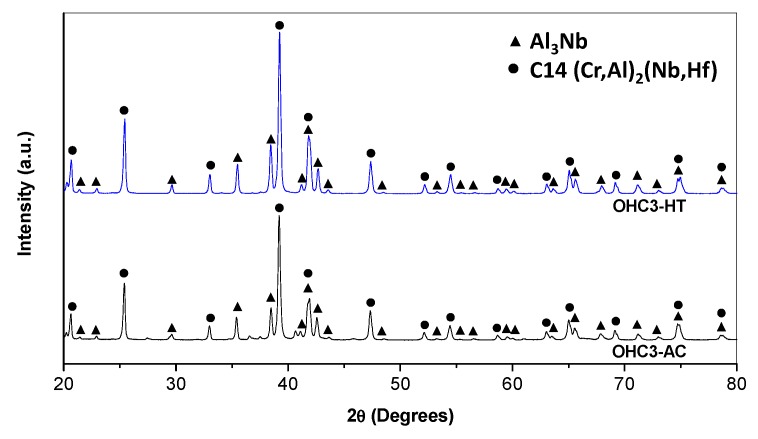
Powder X-ray diffractograms of the alloy OHC3 in the cast and heat-treated conditions.

The actual composition (at %) of the cast alloy (OHC3-AC) was Al-26.6Nb-6.3Cr-0.5Hf. This was the average of the analyses taken from all parts of the button. There was weak macro-segregation of Cr, of which the minimum and maximum concentrations respectively were 5.1 and 8 at %. The XRD and EDS data (Figure 1 and Figure 2) confirmed the presence of four phases, namely the NbAl_3_, the C14 Laves (Nb,Hf)(Al,Cr)_2_ (hereafter the Laves phase), the (Cr, Al, Nb)_ss_ (hereafter the solid solution), and an Hf-rich phase. The latter two were observed near the interface of the NbAl_3_ and Laves phases and were formed at very low volume fractions. The Hf-rich particles were distributed randomly. The solid solution was not detected by XRD owing to its very low volume fraction.

**Figure 3:**
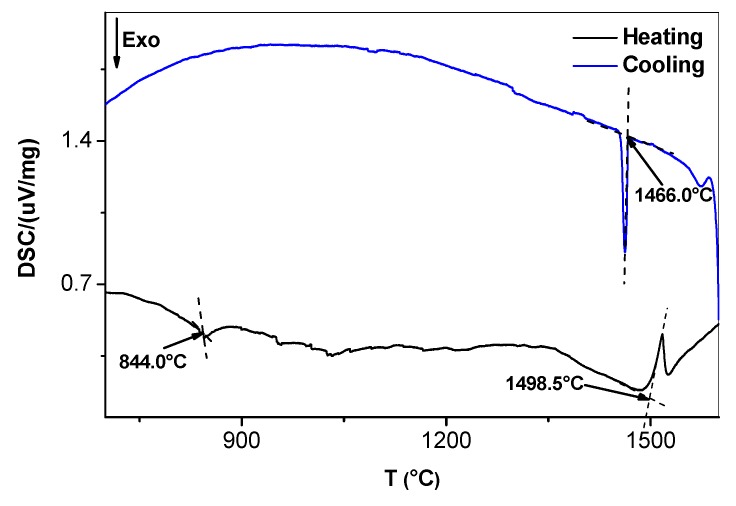
DSC (Differential Scanning Calorimetry) traces showing thermal events on heating and cooling of the alloy OHC3-AC.

The typical microstructure of the alloy OHC3-AC is shown in Figure 1. The average compositions of the phases were as follows: NbAl_3_-73.2(0.5)Al-25.7(0.4)Nb-0.9(0.2)Cr-0.2Hf, Laves phase-46.1(0.8)Al-32.1(0.6)Nb-20.6(0.9)Cr-1.1(0.2)Hf, solid solution-45.4(0.8)Al-7.3(1)Nb-46.7(1.6)Cr-0.6Hf, where in parentheses the standard deviations are given. The microstructure in the top and bulk of the button was essentially the same and consisted of primary NbAl_3_ surrounded by a halo of the Laves phase and a eutectic (Figure 1a) of the Laves and the NbAl_3_ phases. The average compositions of the eutectic and its phases were as follows: eutectic-53.1(0.5)Al-30.3(0.3)Nb-15.7(0.7)Cr-0.9Hf, NbAl_3_-70.7(1)Al-25.5(0.4)Nb-3.5(1.1)Cr-0.3Hf, Laves phase-45.5(0.7)Al-31.6(0.3)Nb-21.3(0.9)Cr-1.6(0.1)Hf. The eutectic was not observed in the bottom of the button. In the latter, the Laves phase was formed at a higher volume fraction. The Hf-rich phase particles were also rich in Cr (Figure 1c). Their shape was similar to HfO_2_ particles formed in Nb-silicide based alloys owing to the scavenging of oxygen by Hf. In Figure 1c it is possible to observe some Cr enrichment in the solid solution areas. In the DSC trace (Figure 3) there was an exothermic signal at 845 °C. There was also a thermal event on heating with peak temperature ~1517 °C (Figure 3) and a thermal event on cooling at 1466 °C.

**Figure 4:**
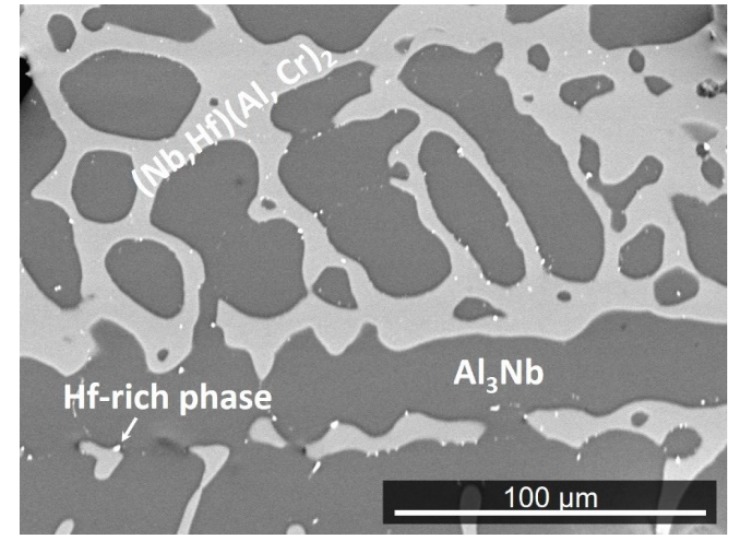
BSE image of the microstructure of OHC3-HT, X640.

The actual composition of the heat-treated alloy (OHC3-HT) (1400 °C/100 h) was Al-26.7Nb-7.0Cr-0.5Hf. Figure 4 shows the typical microstructure, which comprised of three phases, namely the NbAl_3_, Laves phase, and Hf-rich particles. The average compositions of the two compounds were NbAl_3_-73.5(0.2)Al-25.6(0.1)Nb-0.8Cr-0.1Hf, and Laves phase-43.2(0.5)Al-32.1(0.4)Nb-23.3(0.8)Cr-1.5(0.2)Hf. The XRD confirmed the presence of the former two (Figure 2). The solid solution was not present. The two compounds had formed a co-continuous microstructure in which the Laves phase had become coarser. The volume fraction and size of the Hf-rich particles had increased.

### 4.2. Isothermal Oxidation

**Figure 5:**
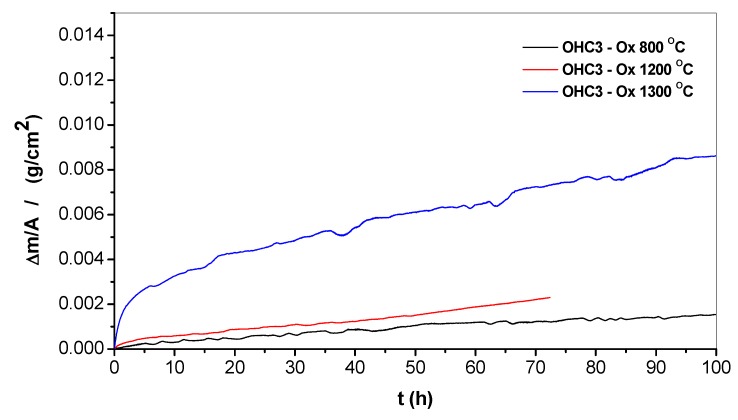
Mass change versus time data for isothermal oxidation at 800 °C, 1200 °C, and 1300 °C.

The mass changes for each temperature are given in Table 1. The thermo-gravimetric analysis (TGA) data (Figure 5) was analysed using the Equation (1),

(1) ln(Δw) = lnK + nlnt 

where $\Delta w\;=\;\frac{\Delta m}{A}$ and Δw is the mass change per unit area, Δm is the mass change, A is the surface area before exposure, K is the reaction rate constant, and t is the exposure time. The oxidation kinetics is regarded as linear (n = 1), parabolic (n = 0.5), sub-parabolic, or cubic (n ≤ 0.3). If there was more than one oxidation mechanism, the oxidation kinetics of the corresponding section was evaluated using the Equation (2) for linear oxidation and Equation (3) for parabolic oxidation,

(2) $$\Delta {w\;=\;k}_{l}.\;t\;$$

(3) $${\left(\Delta w\right)}^{2}{\;=\;k}_{p}.t\;$$

where k_l_ is the linear rate constant and k_p_ is the parabolic rate constant [70].

The n values and oxidation rate constants are shown in Table 1. The oxidation at 800 and 1200 °C was parabolic, and at 1300 °C was sub-parabolic. The latter is related to a very short initial period in which the alloy gains weight at a very high oxidation rate and then stabilises into a very low parabolic oxidation rate. At 1200 °C there was a change in parabolic rate which slightly increased after the first 43 hours (Table 1).

#### 4.2.1. Oxidation at 800 °C

**Figure 6:**
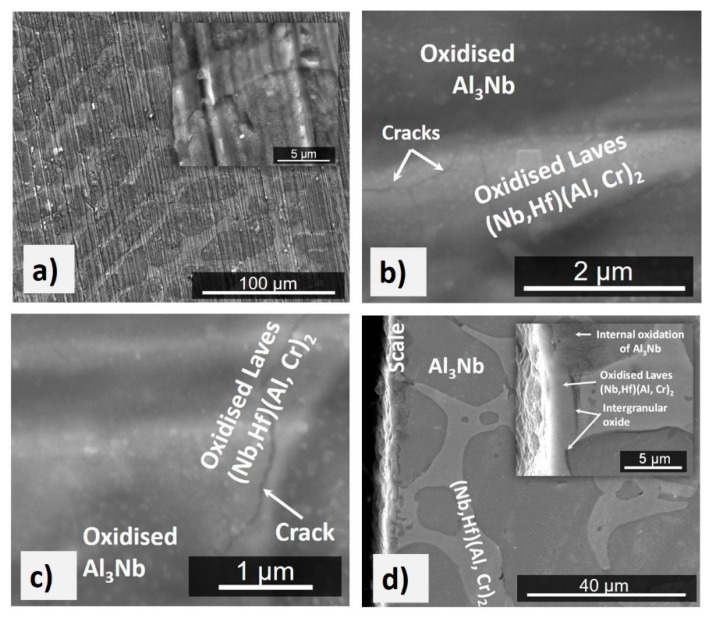
Secondary electron (SE) images of the scale formed on the alloy OHC3 after isothermal oxidation in air at 800 °C for 100 h, (**a**) to (**c**) taken for the oxide surface, (**a**) X1000, (**b**) X70000, (**c**) X 60000, and (**d**) cross-section image, X3000. (**a**) Shows the polishing scratches (parallel “lines”) on the specimen surface that resulted from specimen preparation prior to oxidation.

The mass change was 1.54 mg/cm^2^ (Figure 5). Despite the oxidation temperature being within the pest oxidation regime for the NbAl_3_ phase [71], no pest oxidation had occurred. The specimen had retained its sharp edges and there was no evidence of liquation. The scale was adherent and composed of a black continuous thin film (Figure 6a) that consisted of different oxides depending on the underlying phase in the alloy. The insert in Figure 6a shows a lumped scale on top of the NbAl_3_ phase that was composed of a dark glassy-like oxide with some embedded bright particles. The oxide grown on the top of the Laves phase was flatter and exhibited brighter contrast. Over the NbAl_3_/Laves phase interfaces the scale was different from that formed over each individual phase. The scale on top of the NbAl_3_ was coarser than that on top of the Laves phase which was more compact (Figure 6d). There was also internal oxidation. This was observed in both phases and in the phase boundaries near the alloy/scale interface. Along the phase boundaries, the internal oxidation was about 12 µm depth below the alloy/scale interface (Figure 6d).

**Figure 7:**
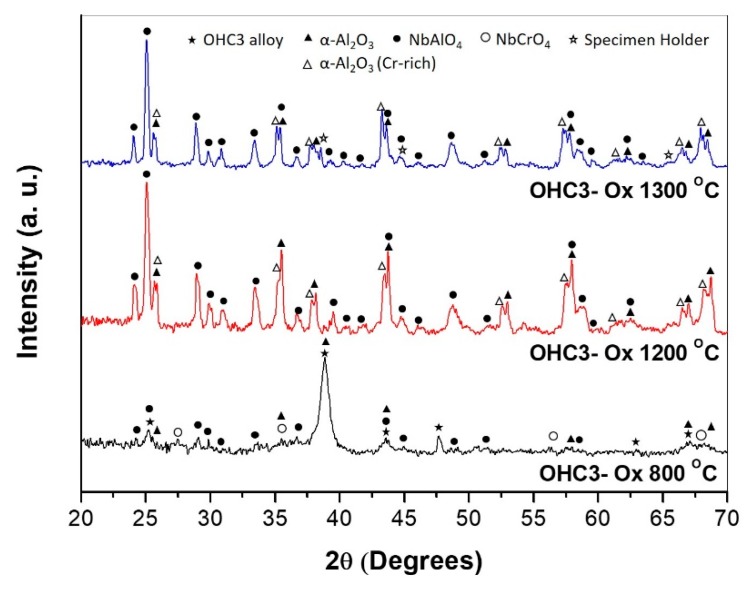
Glancing angle (θ = 2°) X-ray diffraction (XRD) data of the scales formed in air at 800 °C, 1200 °C, and 1300 °C.

In the GXRD data (Figure 7) some peaks from the alloy (substrate) were still visible due to the small-scale thickness. These peaks were from the NbAl_3_ compound (JCPDS 65-7073) and the C14-NbCr_2_ Laves phase (JCPDS 03-9845). The oxide peaks were for αAl_2_O_3_ (JCPDS 10-0173), CrNbO_4_ (JCPDS 30-0366), and AlNbO_4_ (JCPDS 41-0347). The EDS analyses revealed that the scale formed over the NbAl_3_ phase was composed of an outer layer of (Al,Cr)NbO_4_ and an inner layer of αAl_2_O_3_ and the scale formed on top of the Laves phase was composed of an outer layer of (Cr,Al)NbO_4_ with an inner layer of (Cr,Al)_2_O_3_.

**Figure 8:**
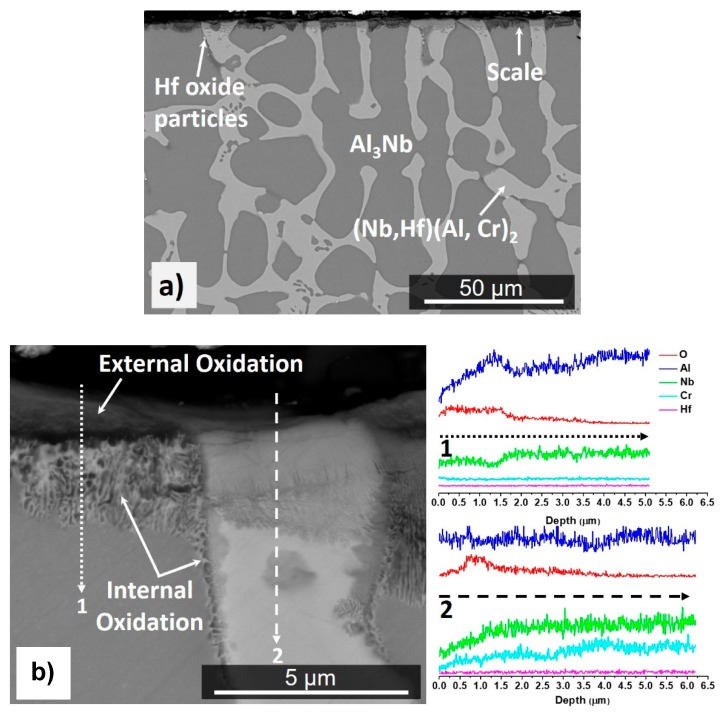
BSE images of cross-sections of the alloy OHC3 after isothermal oxidation in air at 800 °C for 100 h (**a**) X1600, (**b**) elemental line scans (BSE image X24000).

The elemental line scans in Figure 8b show the elemental distribution along two lines of analyses starting from the alloy (substrate)/scale interface. Considering the GXRD data (Figure 7) and the EDS line scan number 1 from the top of the scale to the NbAl_3_ below the internal oxidation zone (IOZ), there was an external layer of (Nb,Cr)AlO_4_ with Al enrichment at the alloy/scale interface and an inner Al_2_O_3_ layer. There was depletion in Al near the alloy/scale interface where the Al was preferentially oxidised. Below the IOZ the Al, concentration in the NbAl_3_ did not change (70 at % Al) but the NbAl_3_ was leaner in Al by 3 at % compared with the cast alloy. It was not possible to analyse the bright contrast phase within the IOZ. The EDS line scan number 2 is over the Laves phase, starts with the (Cr,Al)NbO_4_ at the surface of the scale, and shows Al_2_O_3_ as the internal oxide. In the Laves phase, the bright areas were Cr-rich and the areas with a dark grey contrast were Al-rich. The phase boundaries were rich in Cr.

#### 4.2.2. Oxidation at 1200 °C

**Figure 9:**
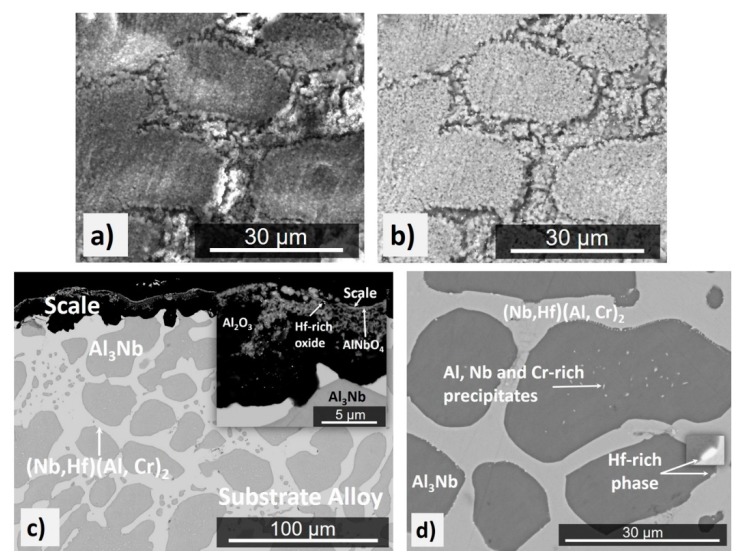
The scale and microstructure of the alloy OHC3 after isothermal oxidation at 1200 °C for 80 h. (**a**) and (**b**) images of the surface of the scale (**a**) SE, X4000, (**b**) BSE, X4000. (**c**) and (**d**) BSE images of cross-sections, (**c**) shows the scale composed of two species, X1000, insert X8000 (**b**) the microstructure of the substrate, X4000.

The mass change per unit area was 4.47 mg/cm^2^ (Table 1). The scale showed good adherence. Some spallation of the external scale occurred during the handling of the specimen. The scale was composed of a continuous oxide that formed on top of the NbAl_3_ phase and was different from that which had formed over phase boundaries and over the eutectic and the Laves phase, where mixed oxides were observed (Figure 9a,b).

The GXRD data (Figure 7) confirmed the presence of αAl_2_O_3_ (JCPDS 10-0173), Cr-doped Al_2_O_3_ (JCPDS 71-1123), and AlNbO_4_ (JCPDS 41-0347) in the scale. The cross-section image in Figure 9c shows that the scale was composed of two parts (layers), the outer part (bright grey contrast) was (Al,Cr)NbO_4_ mixed with Al_2_O_3_ particles (dark grey contrast) and Hf-rich oxide particles, and the inner part consisted of Al_2_O_3_. The Al_2_O_3_ layer beneath the outer part was adherent and formed continuously all over the substrate. The scale had lumps over phase boundaries and the inner Al_2_O_3_ layer was able to withstand the stresses (without buckling) that resulted from the oxidation of the eutectic. Hf-rich oxide particles were also observed in the oxidized Laves phase.

The scale thickness was in the range 7 to 32 μm. Over the areas where the eutectic was absent in the cast microstructure, the thickness of the scale that formed over the NbAl_3_ and Laves phases was the same. Over the eutectic areas, the scale was thicker owing to the large number of phase boundaries that allowed a faster inward diffusion of oxygen. The growth of the oxide over the grain and phase boundaries would have increased the stresses that would have caused the scale to deform and some cracks to form in the Al_2_O_3_ layer. Pores were observed over the Laves phase at the alloy/scale interface but no scale buckling. In the substrate, the solid solution was not found (Figure 9c). Some precipitates that were rich in Al, Cr and Nb had formed in the NbAl_3_ (Figure 9d).

The cross-sections and EDS analyses suggested that the scale had grown via simultaneous oxidation of the components in the substrate alloy since all the elemental components were found at the top of the scale in the (Al,Cr)NbO_4_ oxide. The Cr concentration increased towards the surface of the scale, thus Cr_2_O_3_ could have also be formed and then CrO_3_ evaporated at 1200 °C.

#### 4.2.3. Oxidation at 1300 °C

**Figure 10:**
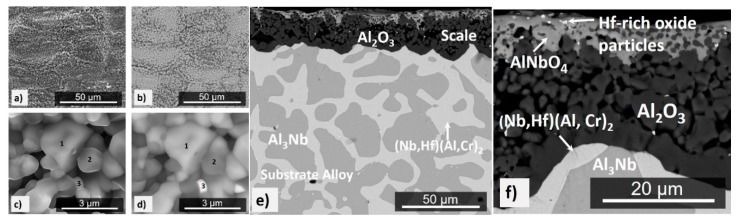
The alloy OHC3 after isothermal oxidation in air at 1300 °C for 100 h. (**a**) to (**d**) scale surface, (**a**) SE image, X2500, (**b**) BSE image, X2500, (**c**) SE image, X40000, and (**d**) BSE image, X40000. (**d**) and (**f**) BSE images of cross-sections (**e**) X1000, and (**f**) X6000. For the numbers 1 to 3 in (**c**) and (**d**) see text.

The mass change per unit area was 8.5 mg/cm^2^, see Table 1. The oxidised specimen presented sharp edges, lumped corners, and some oxide spallation that did not expose the alloy (substrate) but only the inner scale layer (see below). The scale was continuous with some bulging over the Laves phase where mixed oxides were formed. There was porosity in these areas but no cracks (Figure 10a,b).

The morphology of the oxides in the scale is shown in Figure 10c,d. These images were taken near the Laves phase areas where all the oxide particles could be captured in one image. Qualitative EDS analyses were performed on the oxide particles numbers 1 to 3 of which the main elements were (Al, Nb, Cr, O) in number 1, (Al, Cr, O) in number 2, and (Hf, Al, Nb, O) in number 3. The particles number 1 and 2 had low Cr concentration, and in the Hf-rich oxide particles (number 3) the intensity of the Hf peak was significantly stronger than that of the other elements. The porosity in the scale can be seen in Figure 10f. It was formed over the Laves phase in the alloy. In the scale, the concentration of Cr was low, which would suggest that CrO_3_ evaporation had contributed to the porosity in the scale.

The scale consisted of Al_2_O_3_ corundum (JCPDS 10-0173) and Cr-doped Al_2_O_3_ (JCPDS 71-1123) and AlNbO_4_ (JCPDS 41-0347) (Figure 7). The scale was composed of a bright contrast thin outer part (layer) and a thick inner part (dark contrast) with porosity in the coarser areas. The scale thickness was in the range 20 to 50 μm. The outer part thickness was in the range 3 to 15 μm and it was a mixture of AlNbO_4_, Al_2_O_3_, and Hf-rich oxide particles. At the alloy/scale interface, the Al_2_O_3_ (dark contrast) was continuous, compact, and adherent and some Hf-rich oxide particles were also present (Figure 10e,f). There was a small depletion of Al in the NbAl_3_ near the alloy/scale interface where the Al concentration was reduced from 73 (in the cast alloy) to 69 at % Al. The decrease of the Al concentration in the Laves phase was more significant, from 46 (in the cast alloy) to 38 at % Al. However, the Nb_2_Al was not observed.

The growth of the scale seemed to be affected by surface orientation. Indeed, the scale was thicker in some faces of the cubic specimen, where both compounds (NbAl_3_ and Laves phase) had suffered their highest depletion of Al. The change in the Al content of the two compounds was different in the faces of the cubic specimen where the scale was thinner. In these areas the Al content in the NbAl_3_ had hardly changed (the quantitative EDS showed increase from 73 to 75 at % Al) while in the Laves phase, the Al content had decreased slightly from 46 to about 42 at %. Given that the solid solution was formed at a very low volume fraction in the cast alloy, it is possible that there were differences in the solid solution content below different faces of the cubic specimen where the solid solution had served as an effective Al reservoir. It should be noted that the solid solution was not present in the microstructure after the oxidation at 1300 °C.

## 5. Discussion

### 5.1. Microstructures

Considering the ranking of the two un-alloyed intermetallic phases and pure Cr in terms of their melting temperatures, the data from the phase diagrams is T_m_^Al3Nb^ = 1714 °C, T_m_^C14-NbCr2^ = 1730 °C, and T_m_^Cr^ = 1863 °C [32]. The concentrations of Cr and Hf in NbAl_3_ were extremely low, meaning the above temperature should not be expected to be affected significantly by these elements. Indeed, using the chemical composition data for NbAl_3_ (see Section 4.1) and the Nb-Al-Cr liquidus projection in [45], the liquidus of this intermetallic should be slightly above 1700 °C. Aluminium has a strong effect on the melting temperature of the C14-NbCr_2_ Laves phase, which decreases significantly with Al in solution [45]. The melting temperature of the Laves phase in OHC3 was about 1550 °C [45].

In the Nb-Cr and Cr-Al binaries [32], the liquidus temperatures of the solid solutions (Cr-7.3Nb)_ss_ and (Cr-45Al)_ss_ respectively, are about 1820 °C and 1580 °C. The liquidus temperature of (Cr-45Al)_ss_ is below 1580 °C in the revised Cr-rich part of the Al-Cr binary by Stein et al. [45]. The solution of Al and Nb in Cr reduced the liquidus temperature of the solid solution in OHC3 to below 1500 °C [45]. Thus, the ranking of the alloyed phases in OHC3 in terms of decreasing temperature is T_m_^Al3Nb^ (≈ 1700 °C) > T_m_^(Cr,Al,Nb)^ (≈ 1580 °C) > T_m_^C14-(Nb,Hf)(Cr,Al)2^ (≈ 1550 °C). The cast microstructure (Figure 1) would suggest that the NbAl_3_ was the primary phase and that the Laves phase formed the eutectic with the NbAl_3_. The solid solution was formed at a very low volume fraction close to the eutectic. The primary NbAl_3_ is in agreement with the above ranking of the three phases and the Nb-Al-Cr liquidus projection proposed by Stein et al. [45]. The solidification path of the alloy was L→L + NbAl_3_→NbAl_3_ + (NbAl_3_ + C14-(Nb, Hf)(Cr, Al)_2_)_eutectic_ + (Cr, Al, Nb)_ss_ with the NbAl_3_ + C14-(Nb, Hf)(Cr, Al)_2_ eutectic forming between the NbAl_3_ grains, as shown in Figure 1. The Al and Nb concentrations in the eutectic (see Section 4.1) were in excellent agreement with the values (54.2 at % Al and 31.6 at % Nb) reported for the C14-NbCr_2_ + NbAl_3_ eutectic in the alloy 35 (= Al-29.1Nb-9.5Cr (at %)) in [45].

The DSC trace (Figure 3) showed a peak at 1498 °C. Stein et al. [45] attributed a similar temperature (about 1501 °C) to the ternary eutectic NbAl_3_ + C14-NbCr_2_ + Nb_2_Al. For this reason, we studied the alloy extensively in search of the Nb_2_Al. The latter compound was not found. It is suggested that the (NbAl_3_ + C14-(Nb,Hf)(Cr,Al)_2_)_eutectic_ formed in the alloy OHC3-AC at about 1498 °C. The eutectic was absent in the bottom of the button. Considering that there were no significant differences in the composition of the alloy in the bottom and bulk and top of the button, the absence of the eutectic in the bottom must be linked with solidification conditions at a high cooling rate and/or melt undercooling, that are not unusual in arc-melted intermetallic alloys (see Section 2.6) leading to finer microstructure (Figure 1b) and/or a transition like those from anomalous to normal eutectics due to uncoupled growth of the two eutectic phases [72,73]. Stein et al. [45] reported a solid state phase transformation involving (Cr,Al,Nb) and Al(Nb)Cr_2_ phases in the temperature range from 700 to 900 °C depending on the Al content of the Cr_ss_. In the DSC trace of the alloy OHC3 there was an exothermic peak at 845 °C (Figure 3) that might correspond to the same thermal event.

The solubility of Cr in NbAl_3_ in the cast and heat-treated conditions was in the range 0.7 to 1.2 at % Cr and in the NbAl_3_ phase in the eutectic respectively, in the ranges 2.2–4.9 at % Cr, and 0.6–0.9 at % Cr in the cast and the heat-treated alloy. These values were consistent with the solubility values reported in reference [44,74]. Mahdouk and Gachon [75] reported up to 10.8 at % Cr solubility in NbAl_3_ at 1000 °C, a value considerable higher than the above.

It has been suggested that in the Nb-Cr binary, the C14-NbCr_2_ is a metastable phase [45]. The addition of Al is known to stabilise the C14-NbCr_2_ [50]. The Laves phase in OHC3 was the hexagonal C14 type (Figure 2) with average Al content in the range 43 to 46 at % Al, in agreement with [50,74]. Doychak and Hebsur [44] reported the formation of a ternary compound AlNbCr in NbAl_3_ + xCr (x = 1.2, 2.4, 4.8, 6.8 at %) alloys in which the concentrations of Al and Cr respectively were in the ranges 40.1–46.1 at % Al and 23.7–28.4 at % Cr, i.e., the AlNbCr phase in reference [44] had Al + Cr in the range 66.9 to 74.2 (average 69.8 at %), compared with Al + Cr in the range 65 to 68 (average 65.9 at %) for the C14-Nb(Cr,Al)_2_ reported in reference [74] and the average Al + Cr = 66.6 at % for the Laves phase in OHC3. Doychak and Hebsur [44] gave the space group of AlCrNb as P6_3_/mmm. The space group of C14-NbCr_2_ is P6_3_/mmc.

The (Cr,Al)_ss_ shows up to 45.5 at % Al solubility at about 1320 °C in the Al-Cr binary [32,45] and in the Cr-Nb binary the Nb solubility in (Cr, Nb)_ss_ is about 5.6 at % at 1668 °C [76]. Prymak and Stein [74] reported low solubility of Nb in Cr_ss_ even at high Al contents in Nb-Cr-Al alloys and high temperatures. In the Cr-Hf binary [32], the solubility of Hf in the (Cr,Hf)_ss_ is negligible. The Hf content in the solid solution in OHC3 is consistent with the latter.

Following the heat treatment at 1400 °C for 100 h, only the NbAl_3_ and Laves phases were stable (Section 4.1). This is in agreement with the 1450 °C isothermal section of the Nb-Al-Cr ternary in reference [74] in which the alloy OHC3-HT and the two compounds are in the same tie line inside the two-phase NbAl_3_-C14 NbCr_2_ area. In the 1300 °C and 1150 °C isothermal sections in reference [74] the alloy is either very close to or just in the NbAl_3_-(Cr,Al,Nb)_ss_-C14 NbCr_2_ three-phase area and close to the two-phase NbAl_3_-C14 NbCr_2_ area. In the 1000 °C isothermal section of the Nb-Al-Cr ternary proposed by Ivanchenko [77], the alloy is close to the two-phase NbAl_3_-C14 NbCr_2_ area, and next to the three-phase NbAl_3_-(Cr,Al,Nb)_ss_-C14-NbCr_2_ area. The above data for 1000, 1150, and 1300 °C would suggest marginal and reducing stability of the solid solution at 1200 and 1300 °C. At these temperatures, the reduced stability of the solid solution and the consumption of Al in the formation of the scale resulted to the absence of the solid solution in the microstructures.

### 5.2. Oxidation

#### 5.2.1. Oxidation at 800 °C

The NbAl_3_ suffers from pest oxidation at 800 °C [33,71,78,79]. Tolpygo and Grabke [79] suggested that the diffusion of Al and O is faster in the grain boundaries of pure NbAl_3_. The pesting of NbAl_3_ has been linked with the formation of cracks deep into the intermetallic [71] and the selective oxidation of Al to form Al_2_O_3_ on the grain boundaries. The formation of alumina produces compressive stresses that cause the fracture of the low fracture toughness (1.8 to 2.5 MPam^1/2^) NbAl_3_. The cracks allow deeper penetration of oxygen, and further oxide formation and cracking. The αAl_2_O_3_ was identified as one of the oxidation products formed at 800–850 °C on NbAl_3_ that was slightly richer in Al than the stoichiometric tri-aluminide [71].

In the alloy OHC3, the volume fraction of NbAl_3_ was high but the alloy did not suffer from pest oxidation. It formed an external scale (external oxidation) and an IOZ. The external scale was composed of an outer and an inner part with different oxides according to location. In the former, the AlNbO_4_, CrNbO_4_ and αAl_2_O_3_ oxides were observed, in the latter only the αAl_2_O_3_ was found (Figure 7). The NbAl_3_ formed the AlNbO_4_ (assumed to be a result of transient oxidation) above an inner Al_2_O_3_ layer in contact with the alloy (substrate). The Laves phase formed the CrNbO_4_ with an inner Al_2_O_3_ layer. In some areas of the alloy/scale interface the inner continuous Al_2_O_3_ layer was not formed, instead internal oxidation had occurred where alumina oxide was observed. Internal oxidation had occurred in all facets of the cubic specimen and was deeper along the phase boundaries (Figure 8). The internal oxidation is known to cause embrittlement of the substrate and to play a role in scale spallation. At 800 °C the integrity of the scale on OHC3 was practically intact. Few micro-cracks were observed near the phase boundaries and were healed by an oxide. Souza et al. [80] reported that the solid solution in a NbAl_3_ + Cr_ss_ eutectic alloy was able to sustain Al_2_O_3_ growth at 900 °C acting as an Al reservoir. Owing to the very low volume fraction of the solid solution in OHC3-AC, we did not observe Al_2_O_3_ formation over the solid solution.

The depletion of Al in NbAl_3_ can result to the formation of the Nb_2_Al compound [32]. The latter would affect the physical integrity of the microstructure due to the volume contraction generating micro-cracks that subsequently allow further oxidation leading to the disintegration of the alloy i.e., pesting. We did not confirm the presence of Nb_2_Al in the oxidised specimen. The characteristic microstructure of the NbAl_3_ in the IOZ (Figure 8b) exhibited similar morphology to that of a lamellar eutectic or eutectoid and was similar to that of the directionally solidified NbAl_3_ + Nb_2_Al lamellar eutectic [81]. No liquid phase was formed at 800 °C. As internal oxidation occurred, the phase equilibria changed from quaternary Nb-Al-Cr-Hf to quinary Nb-Al-Cr-Hf-O. It is possible that in the IOZ there was solid state transformation of oxygen contaminated tri-aluminide (NbAl_3_ + O) that led to the formation of oxygen-rich tri-aluminide (NbAl_3_, O), aluminide (Nb_2_Al, O), and alumina according to the transformation path (NbAl_3_ + O)→(NbAl_3_, O) + (Nb_2_Al, O)→(NbAl_3_, O) + (Nb_2_Al, O) + Al_2_O_3_.

The Nb_2_Al could be present in the bulk microstructure at 800 °C because, according to available phase equilibria [50], at low (<1000 °C) temperatures the alloy could be in the three-phase NbAl_3_ + C14-Nb(Cr,Al)_2_ + Nb_2_Al area. An exhaustive study of the alloy using EDS and XRD did not confirm the presence of Nb_2_Al. If this phase were present in OHC3, its volume fraction and size must have been too small to be detected by the experimental techniques used.

The Al depletion in the Laves phase affected the oxidation of the alloy owing to change(s) of the Al activity but did not trigger a phase transformation near the alloy/scale interface, presumably because the C14-NbCr_2_ has a wide Al solubility [50] and the Al content in the Laves phase was high (46.1 at% Al). As the Laves phase became contaminated by oxygen there were changes in its chemical composition, as evidenced by the darker contrast near the alloy/scale interface. Deeper below the alloy/scale interface there was evidence of the formation of other phase(s?) (Figure 8b). The NbCr_2_ is a chromia former and one might have expected to find Cr_2_O_3_ in the scale. This oxide was not found (Figure 7). Instead, Cr was detected in regions where Al_2_O_3_ was formed. What is more notable was to find only αAl_2_O_3_ at a temperature where only the transient alumina are found. It is possible that the Cr promoted the formation of the αAl_2_O_3_ by forming Cr_2_O_3_ that acted as the nucleus for the growth of αAl_2_O_3_ and remained in solution in the oxide. A similar mechanism was proposed by Brumm and Grabke [82] for the oxidation of NiAl-Cr alloys. They observed that Cr promoted an accelerated transformation of transient alumina into αAl_2_O_3,_ in which Cr_2_O_3_ nuclei served as nucleation site for αAl_2_O_3_. This would suggest that a higher density of nuclei would lead to a faster formation of αAl_2_O_3_. The fine grain size of the polycrystalline oxide presented high grain boundary areas that served as diffusion paths enabling faster oxide growth. The formation of rod-like oxides of θAl_2_O_3_ at phase boundaries cannot be discarded [44].

Hf additions have been shown to enhance the scale adhesion in alumina forming alloys, for example see [44,47,78,83,84,85,86]. In the alloy OHC3 there was no oxide buckling. This was attributed to the addition of Hf that acted as reactive element and promoted the formation of Hf-rich particles at the oxide grain boundaries.

Chromium addition in Al-rich Nb-Al alloys would be expected to reduce oxygen solubility and diffusivity (see Section 2.2) and enhance the oxidation resistance of Nb-Cr-Al alloys by increasing the Al activity [44]. The oxygen solubility and diffusivity in the alloy at 800 °C was not reduced to a level low enough to prevent internal oxidation. The synergy of Cr and Al in OHC3 stabilized the Laves phase that surrounded the NbAl_3_ tri-aluminide (Figure 1), suppressed the formation of Nb_2_Al and did not promote the formation of a high-volume fraction of the solid solution, and Hf acted as reactive element and formed fine oxide particles that improved the adhesion of the scale and prevented it from buckling, all of which contributed to the suppression of pest oxidation. There was alumina formation in the phase boundary areas in the IOZ but no cracks were observed. Was the latter the result of solute(s?) segregation at the phase boundaries that enabled the alloy to absorb the strain caused by the growth of oxide(s) in these areas via (a) structural transformation, (b) change in deformation mechanism, and (c) change of mechanical properties? The alloying of intermetallics can have significant effects on their stability and mechanical properties [4,15,87]. In the Laves phase, there was Al enrichment and a decrease in Cr content near the phase boundaries. In this region the NbAl_3_ showed a decrease in Al content and Cr enrichment. The line scan number 2 in Figure 8b suggested that the phase boundaries were rich in Cr. There was segregation of Hf in the phase boundary areas, as evidenced by the presence of Hf-rich particles (Figure 4), and both compounds in OHC3 were alloyed (see Section 4.1). The solubility of Hf in DO_22_-NbAl_3_ was very small (see Section 4.1). The calculations of Jiang et al. suggested improved ductility for metastable cubic NbAl_3_ compared with the stable tetragonal DO_22_-NbAl_3_ [48]. The deformation of Laves phases (synchroshear) is accompanied by chemical rearrangements. Fujita et al. [54] have reported increase of the toughness of C15-(Nb,Hf)Cr_2_ to a maximum value of about 4.5 MPam^1/2^ (an increase of about 250%, albeit starting from a low value) for 5 at % Hf. A first-principles study has shown that when Hf occupies the Nb site in C15-NbCr_2_ improvement of fracture toughness is unlikely [88]. To our knowledge, there is no data about the fracture toughness of Hf alloyed C14-NbCr_2_ Laves phase or the deformation and fracture toughness of C14-NbCr_2_ alloyed with Al, Hf and O.

#### 5.2.2. Oxidation at 1200 and 1300 °C

Above 1000 °C the NbAl_3_ oxidises with quasi-linear behaviour [33,71] because of constant cracking and spallation of its scale. At 1200 °C the NbAl_3_ oxidises according to linear kinetics with growth rates approximately 2 to 3 orders of magnitude greater than for typical αAl_2_O_3_ scales [44]. The oxidation of the alloy OHC3 at the same temperature and at 1300 °C was parabolic (Table 1). The oxidation rate constant at 1200 °C was in good agreement with the value of 6.1 10^−11^ g^2^cm^−4^s^−1^ reported by Hebsur et al. for the Al-22Nb-8Cr (at %) alloy, but one order of magnitude higher than that of the alloy Al-24.5Nb-7Cr-0.5Y (at %), which was 8.9 × 10^−12^ g^2^cm^−4^s^−1^ [43]. At 1300 °C the k_p_ value was one order higher than that at 1200 °C (Table 1) and there was partial oxide spallation. There was no internal oxidation at both temperatures (Figure 9c and Figure 10e).

At both temperatures the scale consisted of two parts (layers), an external layer of Al(Nb,Cr)O_4_, with areas of Al(Nb,Cr)O_4_ + Al_2_O_3_, and the thicker Al_2_O_3_ layer at the alloy/scale interface (Figure 7, Figure 9c, and Figure 10e). The Al_2_O_3_ layer showed cavities that were attributed to the fast growth of the oxide in multiple directions causing strain owing to rapid volume changes. Porosity and cavities were observed beneath the αAl_2_O_3_ that formed on top of the Laves phase at the alloy/scale interface, where evaporation of CrO_3_ and diffusion should have played a role in addition to the growth of the oxides in the scale (see above). A high diffusivity of Cr along the interfaces of NbAl_3_ and Laves phase and the grain boundaries of the Al_2_O_3_ scale would have contributed to the formation of porosity. The losses of Cr via the evaporation of CrO_3_ should be reduced with grain growth in the Al_2_O_3_ layer, reducing the grain boundary area and decreasing the Cr supply in the scale and the evaporation of CrO_3_ oxide.

Both phases in the alloy were able to sustain the Al_2_O_3_ growth at the alloy/scale interface where the scale was thinner over the NbAl_3_. Over the Laves phase, the scale consisted of an outer layer of (Al,Cr)NbO_4_ and an inner layer of Al_2_O_3_, exactly the opposite with the oxidation of unalloyed NbCr_2_ at 1200 °C, which forms a different duplex scale composed of an outer layer of Cr_2_O_3_ and an inner layer of CrNbO_4_ [89,90]. This change in oxidation behaviour has been attributed to the high Al concentration in the Laves phase in OHC3 (46.1 at %). At 1200 °C there were precipitates formed in the bulk of NbAl_3_ grains (Figure 9d) and remnants of the eutectic microstructure (Figure 9c) over which the scale was thicker and with lumped areas at the scale/oxidising atmosphere (gas) interface. The phase boundaries in the eutectic had played a very important role in the oxidation. The oxide protrusions and ridges at the scale/gas interface would indicate that outward Al diffusion through short circuit paths had occurred. Counter-current diffusion of oxygen and aluminium are associated with ridges of the same oxide that extends inward and outward [85]. At 1300 °C, precipitates in the NbAl_3_ grains and remnants of the eutectic microstructure were not observed in the alloy (Figure 10e) and consequently, lumped areas at the scale/gas interface were also absent. The alumina was able to deform avoiding cracking. As was the case at 1200 °C, the αAl_2_O_3_ layer showed cavities that could have resulted from the fast oxide growth in multiple directions in these regions causing strain as a result of rapid volume change.

The αAl_2_O_3_ was able to deform, avoiding cracking and separation from the substrate at both temperatures. Reactive element additions in alloys have been associated with the improvement of alumina scale adherence. At 1200 °C in the areas of good scale adhesion a high content of Hf-rich oxide particles was observed. It is likely that scale spallation would occur after prolonged oxidation because the oxide lumps that formed over the eutectic areas would increase the strain in the scale. Microstructure control via heat treatment to dissolve the eutectic prior to oxidation should improve the adherence of the scale. The presence of Hf-rich oxide particles in the scale at 1300 °C (Figure 10f) had contributed to its improved adherence and ductility, in accordance with the anticipated reactive element effect of the Hf addition.

The αAl_2_O_3_ layer formed at 1300 °C was thicker than that formed at 1200 °C. The oxide at the top of the scale suggested that oxidation at this temperature had started with simultaneous oxidation of all the elemental components in the alloy and with some spallation of the scale. The even thickness of the scale (Figure 10) would suggest that at 1300 °C, the Al activity in NbAl_3_ and Laves phase did not differ significantly. Despite the partial spallation of the Al(Nb,Cr)O_4_ + Al_2_O_3_ layer, the inner Al_2_O_3_ layer did not fail since the substrate was not exposed. After the spallation of the outer oxide layer, the Al_2_O_3_ beneath had Hf-rich particles dispersed over its surface, indicating that Hf had positively influenced the Al_2_O_3_ scale but not the (Al,Cr)NbO_4_ layer adherence.

Considering the thickness of the Al_2_O_3_ layer formed at the alloy/scale interface, the solubility range of the NbAl_3_ and the absence of Nb_2_Al beneath the scale, it is likely that (Nb,Hf)(Cr,Al)_2_ formed at the said interface after a considerable Al depletion, as discussed in reference [44]. Nb-Al-Cr alloys that were oxidised at 1200 °C formed a continuous Cr-rich layer of the AlNbCr phase at the alloy/scale interface that not just formed Al_2_O_3_, but was also able to sustain a steady supply of Al to the oxide scale [44]. At 1200 °C there was no continuous (Nb,Hf)(Cr,Al)_2_ layer beneath the Al_2_O_3_ oxide at the alloy/scale interface. At 1300 °C the layer of (Nb,Hf)(Cr,Al)_2_ beneath the Al_2_O_3_ was more continuous but still did not entirely cover the alloy/scale interface. Compared with the oxidation at 800 °C, the capability of OHC3 to form and sustain a continuous Al_2_O_3_ oxide scale was clearly improved at 1200 and 1300 °C.

## 6. Summary and Concluding Remarks

In this work, we studied the alloy Al-25.5Al-6Cr-0.5Hf in the cast and heat-treated conditions and after isothermal oxidation in air at 800, 1200, and 1300 °C. All the requirements for the selection of the alloy were met and it was confirmed that no Zone A and/or layered microstructure was formed in the cast alloy. The microstructure consisted of the alloyed NbAl_3_ and C14-NbCr_2_ compounds, a eutectic of these two compounds, and very small volume fractions of (Cr,Al,Nb)_ss_ and Hf-rich particles, most likely HfO_2_. The prior eutectic microstructure was stable at T < 1200 °C and the solid solution was not stable at T ≥ 1200 °C. At 800 °C the alloy did not pest, and exhibited external and internal oxidation, with AlNbO_4_, CrNbAlO_4_, and αAl_2_O_3_ in the former, and deeper oxidation along the NbAl_3_/Laves phase boundaries in the latter. At 1200 and 1300 °C there was only external oxidation and the scale consisted of two parts (layers), the outer was (Al,Cr)NbO_4_ intermixed with αAl_2_O_3_ and the inner was continuous αAl_2_O_3_. At all three oxidation temperatures, no Nb_2_Al was observed below the alloy/scale interface. Compared with other silicide and silicide + aluminide intermetallic alloys that could be considered for bond coat applications, the alloy OHC3 had the highest mass change per unit area at 800 and 1200 °C, and scale thickness and parabolic rate constant at 1200 °C.

**Figure 11:**
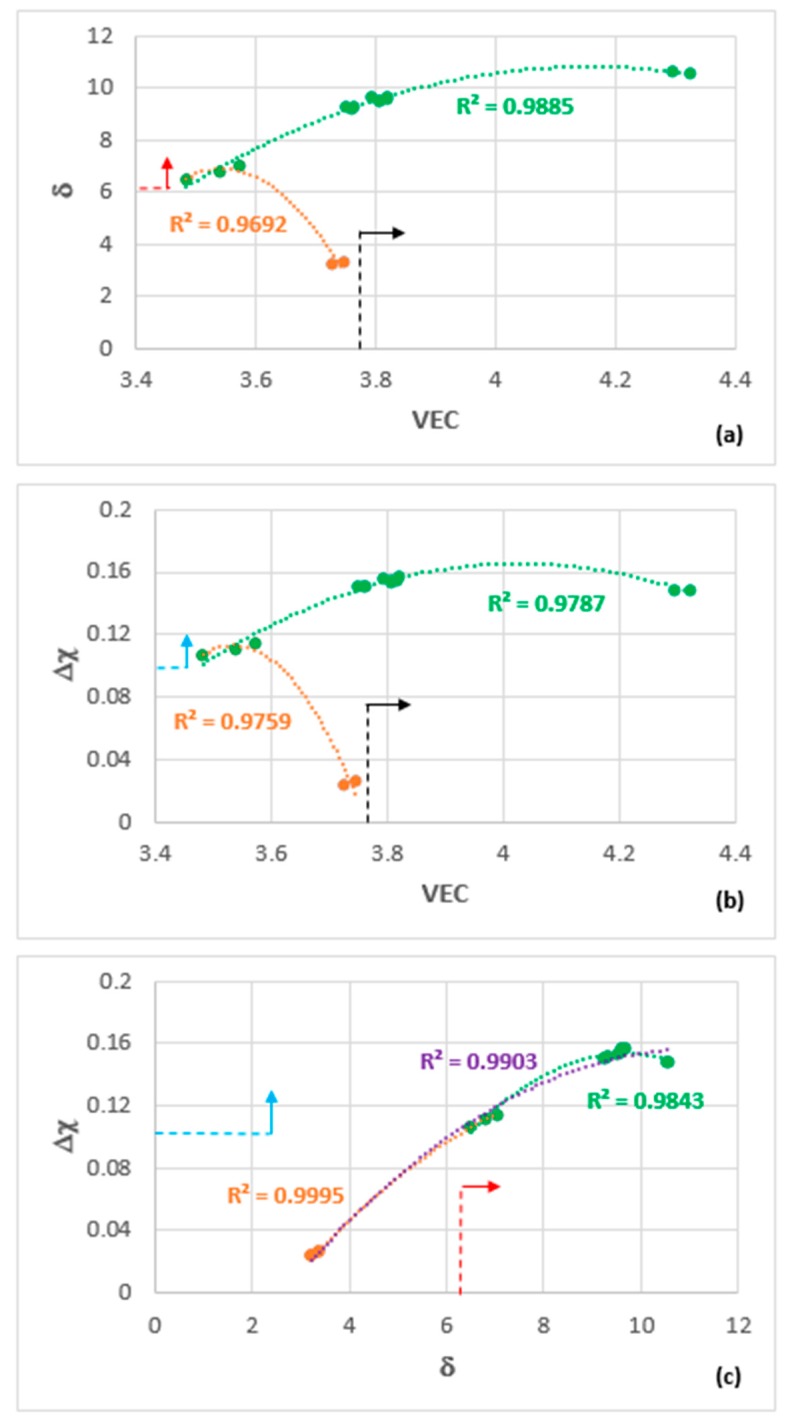
Maps of the parameters δ, Δχ, and VEC (number of valence electrons per atom filled into the valence band). Green colour for the alloys OHC5, MG5, MG6, MG7, Zone A of MG7, orange colour for the alloy OHC3, see also Figure 13 (**a**) δ versus VEC, (**b**) Δχ versus VEC, (**c**) Δχ versus δ. In (**c**) the purple fit (R^2^ = 0.9903) is for all the alloys. For arrows and dashed lines see text.

The alloy OHC3 is shown in the maps of the parameters δ, Δχ, and VEC in Figure 11 where there is parity of colour for the data. The fit of the data of OHC3 with that of the Zone A of the alloy MG7 [21] is very good in all three maps (R^2^ values higher than 0.96), particularly in the Δχ versus δ map, where the fit of all the data, meaning the data for the alloys OHC5 [30], MG5, MG6, MG7 [21,28], Zone A of MG7 [21], and OHC3 (this work) is remarkably good (R^2^ = 0.9903). The parameter VEC can differentiate between the alloys better, as demonstrated in the δ versus VEC and Δχ versus VEC maps in Figure 11.

The selection of the Al concentration in the alloy OHC3 was based on the requirement (c), which was discussed in Section 2, namely the microstructure should consist of primary DO_22_-NbAl_3_ tri-aluminide and C14-NbCr_2_ Laves phase with no stable solid solution (see Section 2) and the desire to find out whether a layered microstructure or Zone A could form (Section 2.6). The requirement (c) has been met but no layered microstructure or Zone A was observed, even though the fit of the data of the alloy OHC3 with that of the Zone A in the alloy MG7 was very good, see Figure 11.

**Figure 12:**
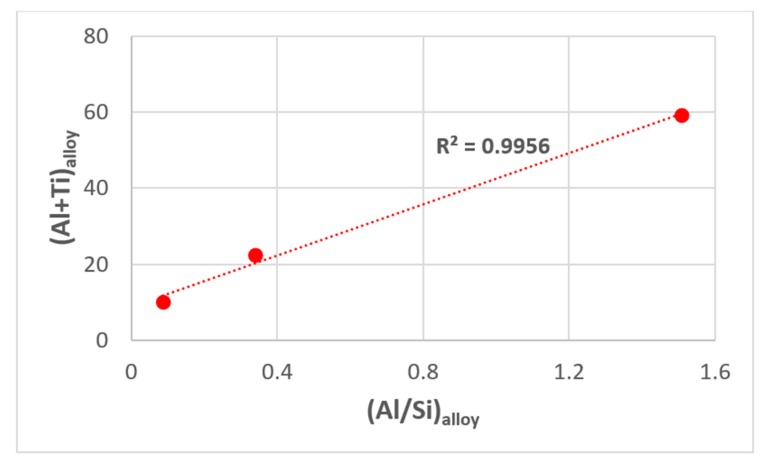
Plot of the relationship between (Al + Ti)_alloy_ versus (Al/Si)_alloy_ for the alloys OHC2, OHC5, and MG7 that form Zone A and/or layered microstructures [21,29,30], see text.

Zone A formation was observed in the alloys MG7 [21] and OHC2 [29] and layered microstructures in the alloys MG7 and OHC5 [30]. Common in all these alloys was the simultaneous presence of Al, Si, and Ti and macro-segregation. In the two alloys where Zone A was formed, the latter was significantly richer in Al than the rest of the cast alloy [21,29]. Layered microstructure was observed in the alloy OHC5 [30], where no Zone A formed, and in the alloy MG7 [21]. The limited data that we have would suggest that for Zone A formation the requirements are: (a) high Al concentration, (b) simultaneous presence of Al, Si, and Ti, and (c) strong macro-segregation in the alloy (see Section 2.6). If no Si and Ti are present in the alloy (the case of OHC3) then Zone A cannot form even though the alloy is very rich in Al. If the Al concentration is low but Al is present simultaneously with Si and Ti and there is macro-segregation in the alloy, then a layered microstructure can form (the case of alloy OHC5). The aforementioned “inter-dependence” between Al, Si, and Ti (or in other words the synergistic effects of Al, Si, Ti and macro-segregation for formation of Zone A and/or layered microstructure) is clearly shown in (Al + Ti)_alloy_ versus (Al/Si)_alloy_, ((Al + Si)/Ti)_alloy_ versus (Al + Ti)_alloy_ and ((Al + Si)/Ti)_alloy_ versus (Al/Si)_alloy_ maps, of which only the former is shown in Figure 12 where the R^2^ = 0.9956.

If Zone A formation were to be sought then the values of the parameters δ, Δχ, and VEC should be higher than those indicated by the red, blue, and black dashed lines and arrows in Figure 11, namely δ > 6, Δχ > 0.10 and VEC > 3.75. If we consider the upper limit of each of the aforementioned parameters that was shown in Figure 1 in reference [29], we suggest that BC alloys with Al, Cr, Hf, Nb, Si, and Ti as alloying elements should be designed/selected to have the parameters δ, Δχ, and VEC in the as manufactured condition and after exposure to high temperature in the following ranges, 6 < δ < 10.5, 0.10 < Δχ < 0.16 and 3.75 < VEC < 4.5.

**Figure 13:**
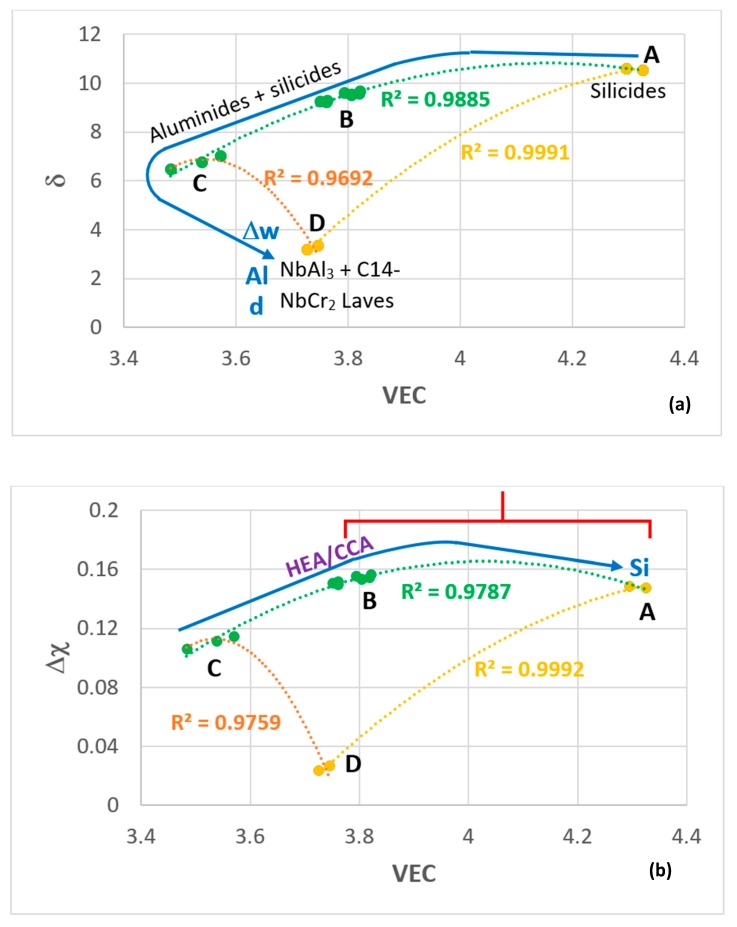

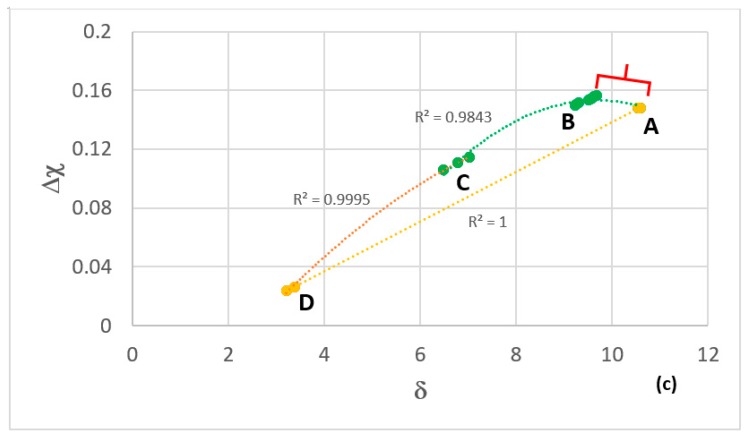
Maps of the parameters δ, Δχ, and VEC with data for the alloys OHC5, MG5, MG6, MG7, Zone A of MG7, and OHC3, see also text, Table 2, and Figure 11. In the maps, area A corresponds to OHC5, B to MG5, MG6, MG7, C to Zone A of MG7, and D to OHC3. The microstructures in different areas of the maps and the “direction” of increase in Al concentration, mass change per unit area (Δw), and oxide thickness (**d**) are shown in (**a**). The area where alloys could be considered as High Entropy Alloys (HEA) or Complex Concentrated Alloys (CCA) and the “direction” of increase of Si concentration in the map are shown in (**b**). The area where the oxidation rates of alloys are in the range of NiAl and Ni-Cr-Al alloys at 1200 °C is shown by the red brackets in (**b**) and (**c**). Notice parity of colours with Figure 11.

The alloys OHC3, OHC5, MG5, MG6, and MG7 are compared in Table 2. In this Table are the nominal compositions of the alloys, the actual composition of the Zone A in the alloy MG7, the phases in the microstructures of the alloys in the cast and heat-treated conditions, the pest behaviour of the alloys, the mass change per unit area at 800 and 1200 °C, the parabolic rate constant k_p_, and the scale (oxides and thickness (d)) at 1200 °C. The areas where these alloys belong in the maps of the parameters δ, Δχ, and VEC are shown by the capital letters A to D in Figure 13, in other words, A is for OHC5, B is for MG5, MG6, and MG7, C is for the Zone A of MG7, and D is for OHC3. The parity of colours in Figure 11 and Figure 13, the remarkable fit of data (R^2^ > 0.999) of the alloys OHC3 and OHC5, and the better separation of the areas indicated by the letters A to D in the δ versus VEC and Δχ versus VEC maps compared with the Δχ versus δ map should be noted.

In Table 2, the concentrations of Al and Si respectively, are shown with bold and/or italics. For example, the concentration of Al increases from the alloy OHC5 to OHC3, and the concentration of Si from the Zone A of the alloy MG7 to OHC5. These trends are shown by the blue arrows in Figure 13a,b respectively, for Al and Si. The increase of mass change per unit area (Δw) and thickness d of scale at 1200 °C from the alloy OHC5 to OHC3 is also indicated by the blue arrow in Figure 13a. The types of intermetallics in the alloys in the different areas of the maps are shown in Figure 13a. In these maps, the nominal compositions of the alloys in the area B meet the “standard definition” of High Entropy Alloys (HEA) (the alloys could also be considered to be Complex Concentrated Alloys (CCA)), see Figure 13b. The k_p_ of typical alumina scales formed on Ni-Cr-Al alloys in air at 1200 °C is about 5.6 × 10^−12^ g^2^/cm^4^·s [44]. The red brackets in Figure 13b,c show the part of the maps where the alumina forming BC alloys with aforementioned alloying elements form alumina scales with rate constants in the range of k_p_ values for NiAl and Ni-Cr-Al alloys [44,91].

To date, the alloying elements that have been considered by our group for possible BC alloys are Al, Cr, Fe, Hf, Nb, Si, and Ti [21,28,29,30] and the alloying with Fe has been identified as not desirable owing to concerns about liquation [29]. The remaining elements are all known to improve the oxidation resistance of Nb-silicide-based alloys [92]. Two more elements are known to improve the oxidation of the latter alloys, namely B and Ge, owing to their effects on phase selection and stability [59,93,94,95]. Furthermore, when the alloying behaviour of Nb-silicide-based alloys is considered, the B-containing alloys form their own group [27]. It is important for future research to find out how B or Ge would affect the oxidation of BC alloys and the location of such alloys in maps of the parameters δ, Δχ, and VEC.

## Figures and Tables

**Table 1:** Mass change, n values and oxidation rate constants of the alloy OHC3 for isothermal oxidation at 800, 1200, and 1300 °C.

Temperature	n	Parabolic Rate Constant K_p_ (g^2^·cm^−4^·s^−1^)	Mass Change (mg/cm^2^)
800 °C	0.68	6.72 × 10^−12^	1.54
1200 °C	0.56	1.78 × 10^−11^ (0–43 h) 5.7 × 10^−11^ (43–80 h)	4.47
1300 °C	0.41	1.88 × 10^−10^	8.5

**Table 2:** Comparison of the alloys OHC3, OHC5, MG5, MG6, and MG7, see also Figure 13.

MAP Area	Alloy [ref]	Alloying Elements (at %)						Phases (AC and HT)	Mass Change (mg/cm^2^) pest Behaviour		k_p_ (g^2^cm^−4^s^−1^)	Scale & Thickness (μm)
		Al	Cr	Hf	Nb	Si	Ti		800 °C	1200 °C	1200 °C	1200 °C
A	OHC5 [30]	5	5		25	60	5	TM_6_Si_5_ C40-TMSi_2_	0.22 no pest	0.85	1.4 10^−12^	αAl_2_O_3_ 5–10
B	MG5 [29]	32.5		3.5	14.5	27	22.5	DO_22_-TiAl_3_ Ti_5_Si_4_, TiAl D8_8_-TM_5_Si_3_ TiSi	0.5 no pest	1.6	8 10^−12^	Niobates αAl_2_O_3_ < 10
	MG6 [29]	37		3.5	13.5	23	23	DO_22_-TiAl_3_ Ti_5_Si_4_, TiAl D8_8_-TM_5_Si_3_ TiSi TM_9_Si_7_Al_4_	0.37 no pest	2.3	3 10^−13^	Niobates αAl_2_O_3_ ≤ 10
	MG7 [21]	35		4	13	24	24	DO_22_-TiAl_3_ Ti_5_Si_4_, TiAl D8_8_-TM_5_Si_3_ TiSi	0.68 no pest	2.6	1 10^−11^	Niobates αAl_2_O_3_ 5
C	Zone A in MG7	54		1.7	11	12.8	20.5	DO_22_-TiAl_3_ D8_8_-TM_5_Si_3_				
D	OHC3	68	6	0.5	25.5			DO_22_-NbAl_3_ C14-NbCr_2_	1.54 no pest	4.47	5.7 10^−11^ −1.8 10^−11^	NbAlO_4_ αAl_2_O_3_ 7–32
